# ADIPOQ polymorphisms and haplotypes affect circulating adiponectin levels and their association with gestational hypertension and preeclampsia

**DOI:** 10.3389/fphys.2025.1736993

**Published:** 2026-01-12

**Authors:** Ricardo Nodari Fróes de Castro, Daniela Alves Pereira, Juliana de Oliveira Cruz, Ana C. Palei, Jackeline S. Rangel Machado, Ricardo C. Cavalli, Jose E. Tanus-Santos, Valéria Cristina Sandrim, Marcelo Rizzatti Luizon

**Affiliations:** 1 Department of Genetics, Ecology and Evolution, Institute of Biological Sciences, Federal University of Minas Gerais, Belo Horizonte, Minas Gerais, Brazil; 2 Department of Biological Sciences, Federal Center for Technological Education of Minas Gerais (CEFET-MG), Belo Horizonte, Minas Gerais, Brazil; 3 Department of Biophysics and Pharmacology, Institute of Biosciences, Universidade Estadual Paulista (UNESP), Botucatu, Brazil; 4 Department of Biological Sciences, State University of Santa Cruz (UESC), Ilhéus, Bahia, Brazil; 5 Department of Surgery, University of Mississippi Medical Center, Jackson, MS, United States; 6 Department of Gynecology and Obstetrics, Ribeirao Preto Medical School, University of Sao Paulo, Ribeirao Preto, Brazil; 7 Department of Pharmacology, Ribeirao Preto Medical School, University of Sao Paulo, Ribeirao Preto, Brazil

**Keywords:** adiponectin, ADIPOQ, biomarkers, genetic polymorphisms, gestational hypertension, haplotypes, preeclampsia

## Abstract

**Introduction:**

Imbalance of adipocytokines has been implicated in endothelial dysfunction in hypertensive disorders of pregnancy (HDP). Adiponectin is an adipocytokine that regulates metabolism, insulin sensitivity, and inflammation, and increased adiponectin levels have been associated with mortality in subjects with cardiovascular diseases. Adiponectin also plays a role in trophoblast invasion during placental development. *ADIPOQ* gene has polymorphisms that modulate adiponectin levels and are linked to several diseases, including gestational hypertension (GH) and preeclampsia (PE). However, no previous study has examined whether *ADIPOQ* SNPs and haplotypes affect adiponectin levels in HDP.

**Objectives:**

We assessed and compared plasma adiponectin levels among healthy pregnant (HP, n = 182), GH (n = 121), and PE (n = 133) women, and examined whether *ADIPOQ* SNPs rs266729, rs2241766 and rs1501299, and their haplotypes are associated with susceptibility to HDP; and whether these polymorphisms and haplotypes affect adiponectin levels in HP, GH and PE.

**Materials and Methods:**

Adiponectin concentrations were determined using the Human Adiponectin ELISA kit. Genotypes were determined by Taqman allele discrimination assays. Haplotype frequencies were estimated using Haplo. stats. Multiple logistic/linear regression analyses were adjusted for selected covariates.

**Results:**

Plasma adiponectin levels were higher in PE than in HP, including when patients were stratified by body mass index. Regarding rs266729 (C>G) SNP, the GG genotype was associated with risk for GH, and the CG genotype may protect against PE. PE patients carrying the CG and GG genotypes showed higher adiponectin levels than their CC counterparts. The ‘G,T,G’ haplotype showed higher adiponectin levels in PE than in HP, and the ‘C,T,T’ haplotype may protect against GH.

**Conclusion:**

Our novel findings indicate that *ADIPOQ* polymorphisms and haplotypes may affect circulating adiponectin levels and the susceptibility to GH and PE.

## 1 Introduction

Hypertensive disorders of pregnancy (HDP) are leading causes of maternal and perinatal mortality and morbidity, and the prevalence of gestational hypertension (GH) and preeclampsia (PE) in worldwide epidemiological studies are 1.8%–4.4% and 0.2%–9.2%, respectively (Umesawa and Kobashi, 2017). PE is characterized as new-onset hypertension defined as systolic blood pressure ≥140 mmHg and/or diastolic blood pressure ≥90 mmHg occurring after 20 weeks of gestation, which are accompanied by proteinuria or other key organ injury (ACOG Gestational Hypertension and Preeclampsia: ACOG Practice Bulletin Number 222, 2020; Magee et al., 2022).

Abnormal development of placenta is closely linked to the pathophysiology of PE (Folk, 2018). The invasion of cytotrophoblasts into the spiral arteries of the decidua is restricted to the superficial layers, leading to reduced perfusion in the placenta, which is followed by widespread dysfunction of the maternal vascular endothelium (Maynard et al., 2005; Meekins et al., 1994). While the mechanisms responsible for PE are not fully clarified, circulating antiangiogenic factors that are released in response to hypoxic/ischemic placental conditions may contribute to the maternal endothelial dysfunction, as reviewed elsewhere (Tomimatsu et al., 2017).

Adiponectin, a collagen-like adipocytokine regulates metabolism, insulin sensitivity, and inflammation (Mori et al., 2010), also plays a role in proliferation, differentiation, and invasion of trophoblasts during placental development, and enhances the migration of trophoblastic cells in the decidua by balancing the matrix metalloproteinases/tissue inhibitors of metalloproteinases axis (McDonald and Wolfe, 2009). Dysregulated adipocytokines, including adiponectin may be associated with endothelial dysfunction in PE (Mori et al., 2010; Ceron et al., 2023; Luizon et al., 2015). Notably, increased adiponectin levels were significantly associated with elevated risk of all-cause and cardiovascular mortality in subjects with cardiovascular diseases (CVD) (Wu et al., 2014), and as an independent predictor of cardiovascular and all-cause mortality in coronary artery disease patients (Yang et al., 2019). Moreover, adiponectin plasma concentrations range from 3 to 30 μg/mL in healthy adults, corresponding to about 0.01% of total plasma proteins (Goldstein et al., 2009). Once synthesized by cells, it can form low-, medium-, and high–molecular weight complexes (HMW–High Molecular Weight), depending on the post-translational modifications involved, with HMW being the biologically active form of adiponectin and the predominant one in human circulation (Kadowaki and Yamauchi, 2005).

Several Genome Wide Association Studies (GWAS) have identified single nucleotide polymorphisms (SNPs) in the *ADIPOQ* gene as significant contributors to the variability in circulating adiponectin levels (Sarsani et al., 2024). Specifically, SNP rs266729 was associated with cardiovascular and metabolic diseases (Pileggi et al., 2014; Smetnev et al., 2019), and SNP rs1501299 as a low-risk factor for the development of CVD with type 2 diabetes (Sun et al., 2012). Moreover, SNPs rs2241766 and rs1501299 were associated with important clinical manifestations of PE, being the rs1501299 associated with serum adiponectin level (Eleuterio et al., 2013). We have previously found a specific association between the CG genotype of the SNP rs266729 and PE. However, except for one study (Eleuterio et al., 2013), no other previous study has examined whether *ADIPOQ* SNPs and haplotypes affect circulating adiponectin levels in HDP.

In this study, we aimed at comparing plasma levels of adiponectin in healthy pregnant (HP) women with those found in patients with GH and PE. We then examined whether the *ADIPOQ* SNPs rs266729, rs2241766 and rs1501299, as well as the haplotypes formed by combination of their alleles affect plasma adiponectin levels in the HP, GH, and PE groups, and whether they are associated with susceptibility to GH and PE.

## 2 Materials and methods

### 2.1 Study population

Approval to study human subjects was obtained from the Institutional Review Board at the Ribeirao Preto Medical School, University of Sao Paulo (FMRP-USP), during the execution of Projects approved by the Research Ethics Committee of the Hospital das Clínicas of FMRP/USP, and each participant giving signed informed consent. The study enrolled 436 pregnant women at the Department of Obstetrics and Gynecology within the University Hospital of FMRP-USP: 182 healthy pregnant, 121 with GH, and 133 with PE diagnosed according to the American College of Obstetricians and Gynecologists (ACOG Gestational Hypertension and Preeclampsia: ACOG Practice Bulletin Number 222, 2020) and the International Society for the Study of Hypertension in Pregnancy (Magee et al., 2022).

GH was defined as systolic blood pressure ≥140 mmHg and/or diastolic blood pressure ≥90 mmHg, confirmed by two separate readings at least 6 h apart, after 20 weeks of gestation, which normalized within 12 weeks postpartum. PE was defined as GH along with considerable proteinuria (≥0.3 g per 24 h) and/or thrombocytopenia, pulmonary edema, impaired liver function, renal insufficiency with abnormal lab values, and new-onset headache unresponsive to all forms of management (ACOG Gestational Hypertension and Preeclampsia: ACOG Practice Bulletin Number 222, 2020; Magee et al., 2022). Individuals with cardiac and renal diseases, diabetes, and those with chronic hypertension, with or without superimposed PE were excluded. Methyldopa was the initial antihypertensive treatment during pregnancy. If the pregnant women did not respond to methyldopa, nifedipine and/or hydralazine were added to achieve targeted blood pressure levels.

Maternal venous blood samples were collected at the time of clinic attendance. Genomic DNA was extracted from the cellular component of 1 mL of whole blood using the salting-out method and stored at −20 °C until analyzed. Plasma samples were obtained after centrifugation of whole blood collected into tubes containing EDTA at 2000 g for 10 min. Those samples were stored at −70 °C until assayed.

### 2.2 Enzyme immunoassays for adiponectin measurement

Plasma adiponectin concentrations were determined using the Human Adiponectin ELISA kit (Human Adiponectin EZHADP-61K, Millipore, St. Charles, MO, United States), following the manufacturer’s instructions, as previously described (Eleuterio et al., 2013).

### 2.3 Genotype determination

Genotypes for SNPs −11377C>G (rs266729; c_2412786_10) in the promoter region, 45T>G (rs2241766; c_26426077_10) in exon 2, and 276G>T (rs1501299; c_7497299_10) in intron 2 of the *ADIPOQ* gene were determined using TaqMan allelic discrimination assays (Applied Biosystems, Foster City, CA, United States). Genotyping was performed in a Real-Time System 7,500 (Applied Biosystems, Foster City, CA, United States). Each PCR was carried out in a total volume of 6.74 mL (3.125 mL of 2 x TaqMan Universal Master Mix and 0.156 mL of primer probe (470 nM and 100 mM, respectively), 3.0 mL of DNA (100 ng/mL) and 0.46 mL purified water-free DNAse/RNAse) placed in 96-well plates. DNA with known genotypes was used as positive control and water as negative control in each experiment.

### 2.4 Statistical analysis

Demographics, clinical characteristics, and plasma adiponectin levels of HP, and patients with PE or GH were compared by Student’s unpaired t-test, Mann-Whitney U test, one-way ANOVA, or χ2 as appropriate, and reported as mean ± s. e.m.

Distribution of genotypes was assessed for deviation from Hardy–Weinberg equilibrium using Hardy–Weinberg exact tests for each locus in each group using the web version of Genepop available at https://genepop.curtin.edu.au/genepop_op1.html (Rousset, 2008). A value of *P* < 0.05 was considered significant. Power calculation was performed using QUANTO version 1.2.4 (Gauderman, 2002). Given the sample size of the study, considering the detectable odds ratio of 0.5 for the minor allele frequency of SNP rs266729, which was found to be significantly associated with PE, the power was 0.929, considering an alpha of 0.05.

The effect of ADIPOQ genotypes on plasma adiponectin levels within each group were compared by analysis of normality (Shapiro-Wilk test), followed by Kruskal-Wallis test (not normally distributed variables) and post-hoc Dunn’s Multiple Comparison test. Haplotype frequencies were estimated using *Haplo. stats* package in R (v4.3.1), which computes maximum likelihood estimates of haplotype probabilities (Schaid et al., 2002). The possible haplotypes including the alleles of the *ADIPOQ* SNPs rs266729 (C>G), rs2241766 (T>G) and rs1501299 (G>T) considered in the analyses were: ‘C, T, G’, ‘C, T, T’, ‘G, T, G’ and ‘C, G, G’. We have excluded the haplotypes with frequency lower than 5% from the analysis. Differences in haplotype frequencies were tested using χ2 tests, and a value of *P* < 0.05 was considered significant.

To further examine the effects of *ADIPOQ* haplotypes on adiponectin levels, we have also performed an additional analysis. We compared *ADIPOQ* haplotypes distributions in two groups of HP, GH and PE patients: the lower and the upper groups, which included subjects with the lower and upper values of plasma adiponectin levels distribution, respectively.

Linkage disequilibrium (LD) was assessed by calculating D′ using the Haploview software (version 4.2; http://www.broad.mit.edu/mpg/haploview/) (Barrett et al., 2005). We used data retrieved from the 1,000 Genomes Phase III study for Europeans (CEU, Utah Residents with Northern and Western European Ancestry), East Asians (CHB, Han Chinese in Beijing, China), and Africans (YRI, Yoruba in Ibadan, Nigeria) (Barrett et al., 2005; 1000 Genomes Project Consortium et al., 2010).

The comparison of adiponectin levels between groups was done by analysis of normality (Shapiro-Wilk), followed by identification of outliers by using the ROUT method for outlier identification (Motulsky and Brown, 2006), and then performance of Kruskal-Wallis test (non-parametric) and post-hoc Dunn’s Multiple Comparison test. The same method was applied for the analysis of adiponectin levels concerning each SNP and their genotypes and haplotypes. Also, we further performed regression analysis for the comparison of adiponectin levels adjusting for all significant covariates, namely, BMI, fasting glucose, ethnicity, and gestational age at sampling.

As an additional analysis, we compared ADIPOQ haplotypes distributions in two groups of HP, GH and PE patients: the lower and the upper groups, which were defined by the median of adiponectin levels on each group, including subjects with the lower and upper values of plasma adiponectin levels distribution, respectively.

Logistic regression analysis was performed using the RStudio integrated development environment (IDE) (https://posit.co/products/open-source/rstudio/) to assess the potential confounding influence of each covariate on the relationship between ADIPOQ genotypes in the PE and GH groups. The variables of clinical importance for GH and PE development were included in the multiple logistic regression models. PE and GH were considered as dependent variables. Genotypes of ADIPOQ SNPs, age, ethnicity, body mass index, primiparity, and gestational age at sampling were considered as independent variables.

## 3 Results

The clinical and demographic characteristics of all pregnant women enrolled in this study are shown in Table 1. The HP and PE groups demonstrated a similar self-declared race, whereas there was a higher White prevalence in GH when compared with HP. Current smoking percentage and primiparity presented similar values between groups (all *P* > 0.05). As expected, both PE and GH groups exhibited higher systolic and diastolic blood pressure compared to the HP group (both *P* < 0.05). It is important to note that most of these patients were on antihypertensive medication. Additionally, patients with GH and PE were older than those in the HP group (*P* < 0.05). Higher body mass index (BMI) and fasting glucose levels were observed in the GH and PE groups compared to the HP group, while hematocrit and hemoglobin were higher only in PE (all *P* < 0.05). There was a lower gestational age at delivery (GAD) in the GH and PE groups, along with lower birth weight and gestational age at sampling in PE (all *P* < 0.05) compared to the HP group. Significant proteinuria was detected in the PE group.

**TABLE 1:** Clinical and demographic characteristics for all subjects selected for the study.

Parameters	HP (n = 182)	GH (n = 121)	P value^a^	PE (n = 133)	P value^a^
Age (years)	24.00 (20.00–28.25)	26.00 (22.00–32.00)	**0.0088**	27.00 (22.00–32.00)	**0.0051**
Race (% white)	58.24	77.69	**0.0253**	72.18	0.2104
Current smoking (%)	9.89	13.22	0.4576	6.77	0.4163
Primiparity (%)	46.30	41.32	0.4680	42.11	0.4823
BMI (kg/m^2^)	27.25 (19.30–45.70)	33.04 (19.68–56.22)	**<0.0001**	31.60 (21.67–51.85)	**<0.0001**
Pre-pregnancy BMI (kg/m^2^)	21.63 (17.26–39.74)	29.33 (17.31–49.61)	**<0.0001**	25.13 (16.18–48.41)	**0.0014**
Pre-pregnancy BMI (kg/m^2^)	21.63 (17.26–39.74)	29.33 (17.31–49.61)	**<0.0001**	25.13 (16.18–48.41)	**0.0014**
SBP (mmHg)	110.00 (90.00–140.00)	130.00 (90.00–210.00)	**<0.0001**	140.00 (100.00–200.00)	**<0.0001**
DBP (mmHg)	70.00 (50.00–90.00)	80.00 (58.00–130.00)	**<0.0001**	90.00 (60.00–130.00)	**<0.0001**
HR (beats/min)	80.00 (60.00–150.00)	80.00 (62.00–110.00)	0.2786	80.00 (60.00–120.00)	0.7653
Fasting glucose (mg/dL)	73.50 (54.00–111.00)	78.00 (60.00–117.00)	**0.0391**	77.00 (60.00–130.00)	**0.0161**
Hb (g/dL)	11.80 (2.30–15.00)	11.90 (7.30–15.70)	0.9623	12.20 (3.80–15.80)	**0.0103**
Hct (%)	35.25 (6.00–49.00)	35.70 (22.00–47.00)	0.9021	36.85 (12.50–47.00)	**0.0074**
Creatinine (mmol/L)	0.70 (0.60–0.80)	0.60 (0.40–1.00)	**0.0231**	0.70 (0.40–2.90)	0.4979
Proteinuria (mg/24 h)	ND	153.00 (20.00–291.00)	ND	665.50 (50.00–776.20)	ND
GAS (weeks)	37.00 (20.00–41.00)	37.00 (18.00–43.00)	0.6079	35.00 (14.00–42.00)	**<0.0001**
GAD (weeks)	40.00 (36.00–42.00)	39.00 (35.00–43.00)	**0.0019**	37.00 (19.00–42.00)	**<0.0001**
Newborn weight (g)	3,265 (2,970–4,970)	3,160 (1,360–4,830)	0.0458	2,883 (1,250–4,235)	**<0.0001**

Abbreviations: BMI, body mass index; DBP, diastolic blood pressure; GAD, gestational age at delivery; GAS, gestational age at sampling; GH, gestational hypertension; Hb, hemoglobin concentration; Hct, hematocrit; HP, healthy pregnant; HR, heart rate; ND, not determined; PE, preeclampsia; SBP, systolic blood pressure. Values are the mean ± s. e.m.

^a^ P < 0.05 vs. healthy pregnant group. Bold values are significant.

Although adiponectin levels were not available for all subjects due to lack of plasma availability, clinical, demographic, and genetic characteristics were collected uniformly for the entire cohort, which is standard practice in observational and genetic association studies. Having complete baseline data for all participants allows us to describe the cohort accurately and confirm that those with and without adiponectin measurements do not differ meaningfully.

**FIGURE 1:**
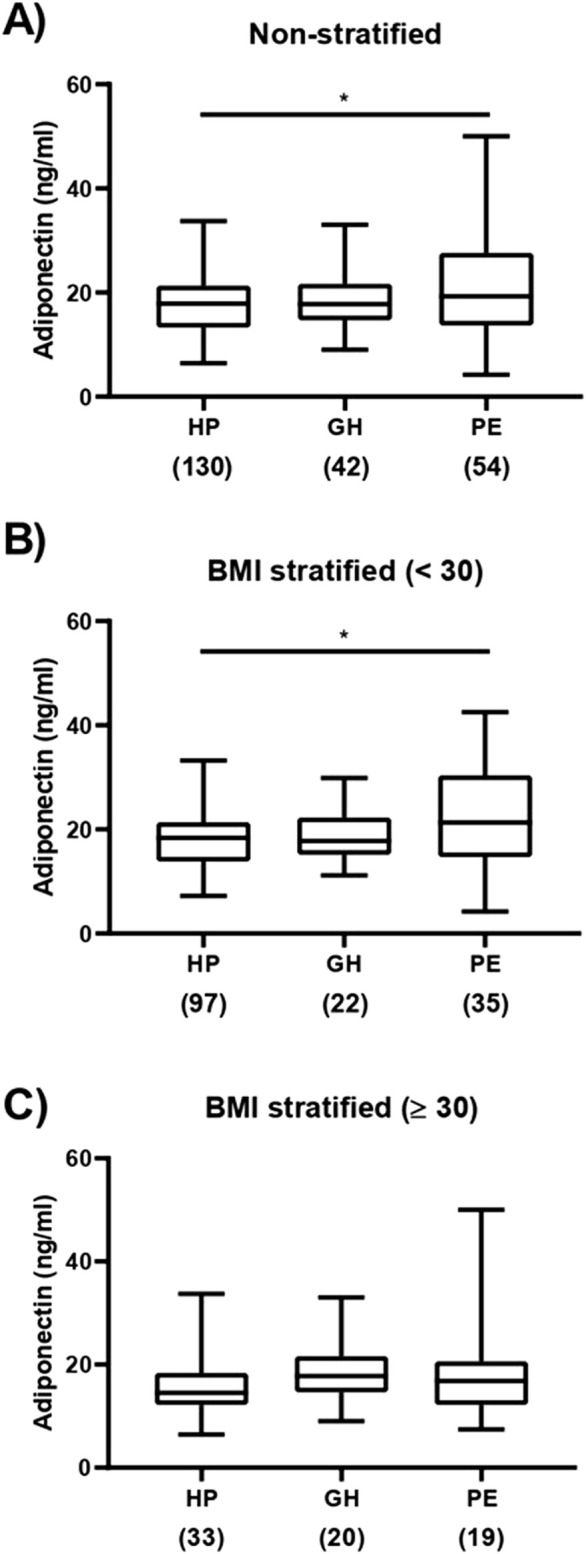
Box-plots of plasma adiponectin concentrations (Median; Minimum–Maximum). In **(A)**, healthy pregnant [HP, N = 130; 17.80 (6.40–33.70)], patients with gestational hypertension [GH, N = 42; 17.75 (9.00–33.00)] and preeclampsia [PE, N = 54; 19.25 (4.20–50,00)]. In **(B)**, [HP, N = 97; 18.40 (7.20–33.20)], patients with GH [GH, N = 22; 17.75 (11.20–29.90)] and PE [PE, N = 35; 21.50 (4.20–42,50)]. In **(C)**, [HP, N = 33; 14.50 (6.40–33.70)], patients with GH [GH, N = 20; 17.75 (9.00–33.00)] and PE [PE, N = 19; 16.80 (7.40–50,00)]. In *P < 0.05 between HP and PE groups. In parentheses below each group, the corresponding group sample size.

The values shown in Figure 1 are for 130 HP women, 42 patients with GH, and 55 patients with PE, the 227 patients that we were able to measure adiponectin levels. The clinical and demographic characteristics for this subset of subjects are shown in Supplementary Table S1. The correlation analysis between *ADIPOQ* polymorphism and serum adiponectin levels was restricted to the 227 patients.

We found significant differences in plasma adiponectin levels between HP and PE (*P* < 0.05; Figure 1A). We further stratified the subgroups of subjects according to BMI <30 and BMI ≥30 kg/m^2^, to examine the influence of body fat in circulating adiponectin levels. While we found increased plasma adiponectin in PE compared to HP for the subgroups with BMI <30 kg/m^2^ (*P* < 0.05; Figure 1B), no differences were found among HP, GH, and PE groups in the subgroups with BMI ≥30 kg/m^2^ (*P* < 0.05; Figure 1C). We further performed a generalized linear regression analysis (Gamma distribution) for the comparison of adiponectin levels adjusting for covariates (Supplementary Table S2). The model showed no significant association between preeclampsia and adiponectin levels (p = 0.1636). BMI, fasting glucose, ethnicity, and gestational age at sampling were also not significant.

The frequencies of *ADIPOQ* genotypes and alleles are shown in Table 2. The distribution of genotypes for each SNP showed no deviation from Hardy-Weinberg equilibrium (P > 0.05), except for the SNP rs266729 (C>G) in the group of patients with PE (P = 0.0009; Supplementary Material S1). Significant departure from Hardy-Weinberg equilibrium can be expected in relatively small samples of patients over a range of genetic models, and it may occur in case groups in case-control association studies (Wittke-Thompson et al., 2005). Considering codominant distribution, the CG genotype for SNP rs266729 was more common in HP compared to PE, while the GG genotype was more frequent in GH compared with HP (both *P* < 0.05; Table 2). Conversely, no differences in genotype and allele frequencies among groups were found for SNPs rs2241766 and rs1501299 (all *P* > 0.05; Table 2). The haplotype distributions are shown in Table 3. The ‘C,T,T’ haplotype was more frequent in HP than in GH (*P* = 0.0397; Table 3). Conversely, no significant differences were found between HP and PE (all *P* > 0.05; Table 3).

**TABLE 2:** Genotypic and allelic frequencies for *ADIPOQ* polymorphisms in healthy pregnancy, gestational hypertension and preeclampsia groups.

*ADIPOQ* SNP	*HP (n = 182)*	*GH (n = 121)*	*OR (95% CI)*	*P value* ^a^	*PE (n = 133)*	*OR (95% CI)*	*P-value* ^a^
rs266729							
Codominant							
CC	106 (58.24%)	64 (52.89%)	1.0000 (Reference)		84 (63.15%)	1.0000 (Reference)	
CG	62 (34.06%)	37 (30.57%)	0.9884 (0.5847–1.6370)	>0.9999	28 (21.05%)	0.5699 (0.3310–0.9658)	**0.0378**
GG	14 (7.70%)	20 (16.52%)	2.3660 (1.1540–4.8160)	**0.0346**	21 (15.78%)	1.8930 (0.8912–4.0140)	0.0986
Dominant							
CC	106 (58.24%)	64 (52.89%)	1.0000 (Reference)		84 (63.16%)	1.0000 (Reference)	
CG + GG	76 (41.76%)	57 (47.11%)	1.2420 (0.7721–1.9620)	0.4083	49 (36.84%)	0.8136 (0.5206–1.2910)	0.4152
Alleles							
C	274 (75.27%)	165 (68.18%)	1.0000 (Reference)		196 (73.68%)	1.0000 (Reference)	
G	90 (24.73%)	77 (31.82%)	1.4210 (0.9931–2.0250)	0.0634	70 (26.32%)	1.0870 (0.7552–1.5520)	0.7110
rs2241766							
Codominant							
TT	146 (80.22%)	88 (72.73%)	1.0000 (Reference)		101 (75.94%)	1.0000 (Reference)	
TG	35 (19.23%)	30 (24.79%)	0.7032 (0.4014–1.2210)	0.2513	27 (20.30%)	0.8968 (0.5066–1.5620)	0.7734
GG	1 (0.55%)	3 (2.48%)	0.2009 (0.0154–1.3730)	0.1575	5 (3.76%)	0.1384 (0.0117–1.0330)	0.0852
Dominant							
TT	146 (80.22%)	88 (72.73%)	1.0000 (Reference)		101 (75.94%)	1.0000 (Reference)	
TG + GG	36 (19.78%)	33 (27.27%)	0.6575 (0.3847–1.1320)	0.1616	32 (24.06%)	0.7783 (0.4576–1.3380)	0.4062
Alleles							
T	327 (89.84%)	206 (85.12%)	1.0000 (Reference)		229 (86.09%)	1.0000 (Reference)	
G	37 (10.16%)	36 (14.88%)	0.6475 (0.4027–1.0450)	0.0973	37 (13.91%)	0.7003 (0.4249–1.1550)	0.1685
rs1501299							
Codominant							
GG	81 (44.51%)	65 (53.72%)	1.0000 (Reference)		67 (50.37%)	1.0000 (Reference)	
GT	82 (45.05%)	47 (38.84%)	1.4000 (0.8621–2.3040)	0.1789	52 (39.10%)	1.3040 (0.8181–2.0980)	0.2801
TT	19 (10.44%)	9 (7.44%)	1.6940 (0.6990–4.1320)	0.2974	14 (10.53%)	1.1230 (0.5320–2.3120)	0.8476
Dominant							
GG	81 (44.51%)	65 (53.72%)	1.0000 (Reference)		67 (50.37%)	1.0000 (Reference)	
GT + TT	101 (55.49%)	56 (46.28%)	1.4470 (0.9055–2.2660)	0.1278	66 (49.63%)	1.2660 (0.8119–1.980)	0.3067
Alleles							
G	244 (67.03%)	177 (73.14%)	1.0000 (Reference)		186 (69.92%)	1.0000 (Reference)	
T	120 (32.97%)	65 (26.86%)	1.3390 (0.9340–1.9230)	0.1258	80 (30.08%)	1.1430 (0.8102–1.6050)	0.4883

Abbreviations: CI., confidence interval; OR., odds ratio.

^a^ P < 0.05 vs. healthy pregnant group. Significant P values are in bold.

**TABLE 3:** Haplotype frequencies for ADIPOQ polymorphisms in the HP, GH and PE groups. The considered haplotypes were the ones with frequency higher than 5% among groups.

*ADIPOQ Haplotypes*	*HP (n = 182)*	*GH (n = 121)*	*P values* ^a^	*OR (95% CI)*	*PE (n = 133)*	*P values* ^a^	*OR (95% CI)*
C.T.G	0.3993 (39.93%)	0.3574 (35,74%)	0.3431	1.0000 (Reference)	0.3667 (36,67%)	0.5205	1.0000 (Reference)
C.T.T	0.2709 (27.09%)	0.1911 (19,11%)	**0.0397**	0.8132 (0.5145–1.2854)	0.2659 (26,59%)	0.6967	1.0463 (0.6991–1.5658)
G.T.G	0.1694 (16.94%)	0.2431 (24,31%)	0.0699	1.4175 (0.8836–2.2742)	0.1935 (19,35%)	0.5779	1.2370 (0.7776–1.9679)
C.G.G	0.0826 (8.26%)	0.1225 (12,25%)	0.1268	1.5546 (0.7932–3.0470)	0.1043 (10,43%)	0.2856	1.3732 (0.7516–2.5088)

*Global-stat = 8.5486*. *df = 5*. *P = 0.12848; Global-stat = 3.7515*. *df = 5*. *P = 0.58572*.

Abbreviations: CI., confidence interval; GH., gestational hypertension; HP., healthy pregnant; OR., odds ratio; PE., preeclampsia.

^a^ P < 0.05 vs. healthy pregnant group. Significant P values are in bold.

**FIGURE 2:**
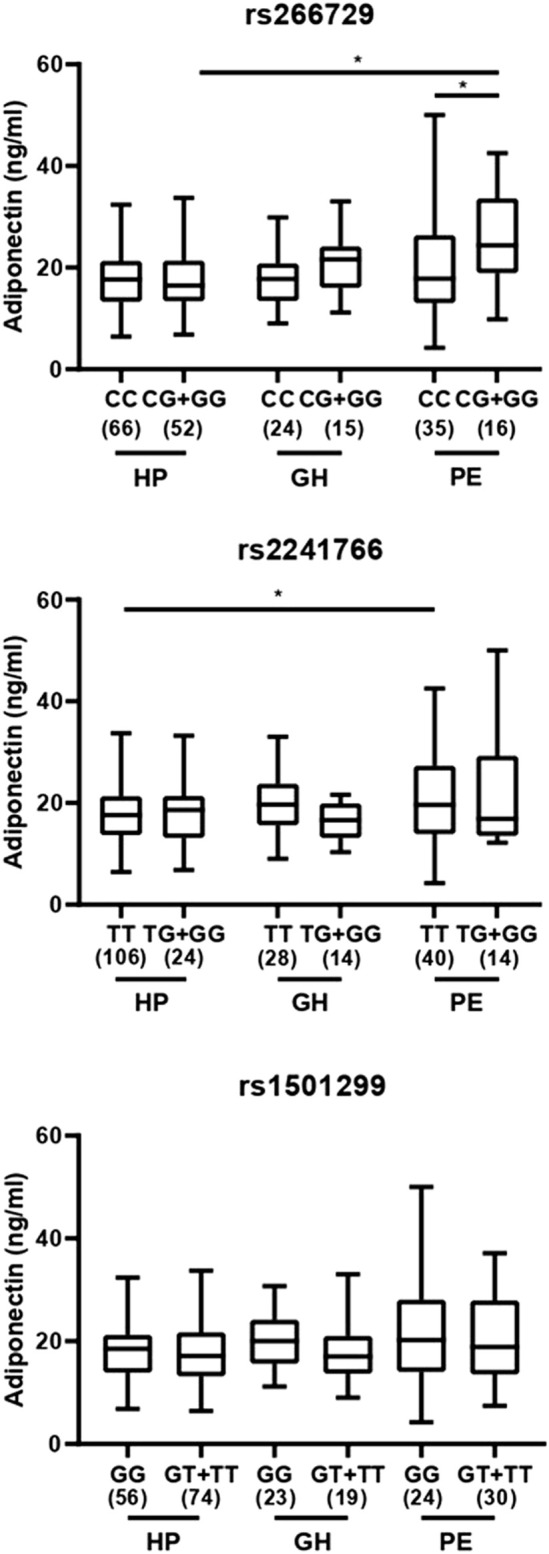
Box-plots of plasma adiponectin concentrations (Median; Minimum - Maximum) in healthy pregnant (HP) women, patients with preeclampsia (PE) and with gestational hypertension (GH) grouped according to the genotypes for three *ADIPOQ* SNPs rs266729 C>G; rs2241766 T>G; and rs1501299 G>T. For each genotype, the sample size is shown in parenthesis. *P < 0.05 for rs2241766 between PE patients with the TT genotype when compared to the same genotype in the HP group. *P < 0.05 for rs266729 between patients with PE carrying CG + GG genotypes versus those carrying the CC genotype, and with HP women carrying the CG + GG genotypes.

**FIGURE 3:**
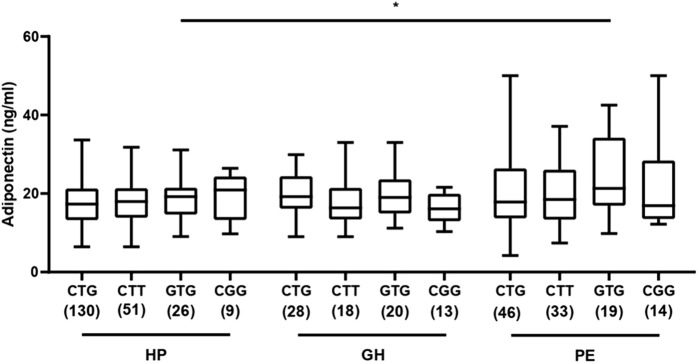
Plasma adiponectin concentrations for each of the most frequent *ADIPOQ* haplotypes (for the three SNPs rs266729 C>G; rs2241766 T>G; and rs1501299 G>T) in healthy pregnant (HP), gestational hypertension (GH), and preeclampsia (PE). For each haplotype, the sample size is shown in parenthesis (sample size considering two haplotypes per patient). *P < 0.05 for comparison between PE and HP groups considering the ‘G,T,G’ haplotype.

We next examined the effect of *ADIPOQ* genotypes and haplotypes on plasma adiponectin levels. We found no difference among groups and within the same group for SNP rs1501299 (all *P* > 0.05; Figure 2). However, we found higher adiponectin levels in patients with PE carrying the TT genotype of SNP rs2241766 (T>G) when compared with TT carriers of the HP group (*P* < 0.05; Figure 2), but no differences between genotypes within each group (*P* > 0.05; Figure 2). Moreover, we found that patients with PE carrying the CG + GG genotypes for SNP rs266729 (C>G) showed higher adiponectin levels than those carrying the CC genotype and CG + GG carriers with HP (both *P* < 0.05; Figure 2). Regarding haplotypes, we found increased adiponectin levels in patients with PE carrying the ‘G,T,G’ haplotype compared to those with the same haplotype in the HP group (*P* < 0.05; Figure 3). Conversely, no differences were found when comparing adiponectin levels between GH and PE nor among haplotypes within the same group (all *P* > 0.05; Figure 3).

In addition, we compared the distribution of *ADIPOQ* haplotypes in the subgroups of lower and upper plasma adiponectin levels for each of the study groups, but found no differences in haplotype frequencies for the HP and GH groups (all *P >* 0.05, Supplementary Tables S3, S4, respectively). For PE, the ‘C,T,T’ haplotype was more frequent in the lower subgroup of adiponectin levels (*P* = 0.0285; Supplementary Table S4), while the ‘G,T,G’ haplotype was more frequent in the upper group of adiponectin levels (*P* = 0.0378; Supplementary Table S5).

We further compared analysis of the influence of covariates performed using univariate and multivariate logistic regression models, evaluating the influence of each variable alone and groups of variables in the outcome for GH and PE. Univariate logistic regression showed age and body mass index during pregnancy independently associated with PE and GH compared to HP women (P < 0.05 and OR>1) and gestational age at sampling primiparity associated with GH compared to HP women (P < 0.05 and OR<1; Supplementary Tables S6, S7).

Logistic regression analysis adjusted for independent variables were done for each of the studied SNPs in GH and PE (Supplementary Table S8; Supplementary Table S9, respectively). For the GH group, the GG genotype of the rs266729 (C>G) SNP influences the GH outcome in the model that considers only the maternal age as a covariate (Supplementary Table S8). On the other hand, the same rs266729 SNP, in the PE group, showed influence in the disease outcome concerning the CG genotype for the multivariate model that has BMI during pregnancy and maternal age as covariates (Supplementary Table S8), and the same result was observed for the models considering maternal age or BMI in pregnancy as the only covariate besides the rs266729 (C>G) SNP (Supplementary Table S5).

Finally, we assessed the pairwise LD among the SNPs rs266729 (C>G), rs2241766 (T>G) and rs1501299 (G>T) in all the studied groups. A short segment of higher LD between SNPs rs2241766 and rs1501299 was found in HP (D’ = 1 and (LOD)’≥2) when compared to PE (D’ = 1 and (LOD)’<2) and to GH patients (D’<1 and (LOD)’<2; Supplementary Figure S1). When analyzing LD of these SNPs in the 1000 Genomes Project populations of European (CEU), African (YRI), Chinese (CHB), and Japanese (JPT) subjects, higher LD blocks were found, particularly in Asian populations (CHB and JPT) (D = 1 and LOD≥2) when compared to CEU and YRI populations (Supplementary Figure S2).

## 4 Discussion

The main findings reported in the present study are: (1) Patients with PE and a BMI <30 exhibited higher plasma adiponectin levels compared to HP women with same BMI range; (2) The CG genotype for SNP rs266729 (C>G) was more frequent in HP women compared to PE patients, while the GG genotype was more frequent in GH patients when compared to HP women; (3) among haplotypes, the ‘C,T,T’ was more frequent in HP than in GH; (4) PE patients carrying the CG + GG genotype for the SNP rs266729 (C>G) had elevated plasma adiponectin concentrations when compared to PE patients with CC genotype and to HP women with the CG + GG genotype, while PE patients carrying the TT genotype for SNP rs2241766 (T>G) presented higher adiponectin levels when compared to the same genotype in HP women; and (5) the ‘G,T,G’ haplotype presented higher adiponectin levels in PE patients when compared to HP women with the same haplotype.

Adiponectin is a protein produced primarily by adipocytes, and has an influence upon metabolism homeostasis, inflammation resolution, and atherosclerosis prevention (Scherer et al., 1995; Song et al., 2016). During normal pregnancy and in patients with normal BMI, plasma adiponectin levels decrease as the pregnancy progresses, especially after the mid-term gestation (Fuglsang et al., 2006). Although circulating adiponectin may be expected to be lower in PE due to its anti-inflammatory effects (Catalano et al., 2006; Sprague and Khalil, 2009), its concentration trends diverge greatly among studies, with some reporting low levels (Hendler et al., 2005; Miehle et al., 2012) and others high levels during PE (Bawah et al., 2020). Despite these conflicting results, these studies suggest a role for adiponectin in the pathophysiology of PE, and our findings of higher circulating adiponectin levels in PE support this concept. Differences found in the results of these studies may be attributed to characteristics of the included population, including ethnicity, lifestyle, and gestational age at sampling. In addition to these factors, we quantified total adiponectin concentration in plasma. However, adiponectin has three different isoforms, being the high molecular weight (HMW) isoform considered the active form (Song et al., 2016). Considering this, further studies are needed to clarify these different findings regarding circulating adiponectin levels in PE.

We found one previous association study on PE and GH that investigated the SNPs rs266729, rs2241766, and rs1501299. This study reported that rs266729 was associated with PE (Machado et al., 2014) Other studies also found associations for rs2241766 and rs1501299 (Youpeng et al., 2010; Saarela et al., 2006). rs1501299 was linked to protection against PE and to changes in serum adiponectin levels. However, no causal relationship was identified between genetically predicted serum adiponectin levels and the risk of PE (Chen et al., 2022). However, the present study considered the codominant and dominant models in the association analysis for genotypes of SNPs rs266729, rs2241766 and rs1501299 with HDP. Noteworthy, when considering the codominant model for the genotypic distribution of SNP rs266729, we found the CG genotype to be more frequent in HP, as a possible protective factor against PE, and the GG genotype as a risk factor of GH. Besides the single-locus analysis, our logistic regression models provided evidence that the association of the CG genotype with PE and the GG genotype with GH for the SNP rs266729 occurred even in the presence of BMI and maternal age as confounding variables. Beyond HDP, the G allele of the SNP rs266729 has been associated with an increased risk of type 2 diabetes mellitus and uncontrolled chronic hypertension (Sun et al., 2017; Cunha et al., 2023). While these findings suggest that carriers of the G allele have an unfavorable outcome, in the present study we found that C and G allelic frequencies were statistically similar in PE or GH versus HP. These discordant findings highlight that further replication association studies in different populations are warranted to confirm our observations.

The examination of LD among the three *ADIPOQ* SNPs for each group revealed that SNP rs266729 is in equilibrium with the other SNPs analyzed in this study, whereas a short segment of higher LD between SNPs rs2241766 and rs1501299 was found in HP when compared to PE and GH. In contrast, when assessing LD of these same SNPs in populations from the 1000 Genomes project, we found high LD blocks, particularly in Asian populations (CHB and JPT). These findings highlight the need to evaluate these SNPs in future studies involving cohorts from diverse ancestries for the validation of the SNP rs266729 as a genetic marker for HDP (Ng et al., 2024).

Regarding the haplotypes, the ‘C,T,T’ haplotype was more frequent in HP than in patients with GH, and may be a possible protective factor against GH. Previous studies focused on haplotypes formed by *ADIPOQ* SNPs in HDP have found an overrepresentation of the pooled G haplotypes versus the TT haplotype for the rs2241766 and rs1501299, respectively, in PE; however, when four different *ADIPOQ* SNPs were considered, the association was lost. Nonetheless, the ‘C,T,T’ haplotype has been associated with risk to metabolic syndrome in a cohort of Sudanese patients (Mosad et al., 2023), highlighting these *ADIPOQ* SNPs as possible targets in further studies.

We observed that PE patients carrier of the G allele (GC + GG genotypes) for SNP rs266729 had higher circulating adiponectin levels when compared to PE patients with the CC genotype, suggesting that this SNP may play a potential role in regulating adiponectin levels in PE. Although previous studies have indicated that serum adiponectin levels are lower in patients with the GG genotype than those with TG + TT genotype for SNP rs1501299 in severe PE, we did not find differences in plasma adiponectin levels between carriers of these genotypes in PE. *ADIPOQ* SNPs have also been shown to affect circulating adiponectin levels in other disease settings. For example, in a subgroup of Chinese subjects, carriers of rs266729 variants were found at high risk of dyslipidemia, atherosclerosis, and coronary artery disease, being the G allele associated with decreased adiponectin (Wang et al., 2022). This may be explained by intrinsic differences between studied populations, but it also could be explained by the allelic and loci heterogeneity (Spracklen et al., 2020), lifestyle differences between subjects, and also the methodology used to measure circulating adiponectin levels.

According to its 1f score in RegulomeDB and ENCODE gene regulation data (Supplementary Figure S3), the rs266729 may affect the binding of transcription factors in the *ADIPOQ* promoter region. Moreover, MethPrimer analysis revealed that the C to G change at SNP rs266729 position (CpG site) creates a potential methylation site (data not shown). Since DNA methylation in the promoter region is linked to gene inactivation or reduced expression (Greenberg and Bourc’his, 2019), this result contrasts with our finding that carriers of the G allele showed higher adiponectin levels in PE. However, it is important to consider that methylation can be altered in pathological conditions such as HDP (Deng et al., 2024). Thus, future studies need to confirm if this CpG site at the *ADIPOQ* locus is indeed methylated in PE.

Furthermore, we noted that PE patients carriers of the ‘G,T,G’ haplotype had higher circulating adiponectin levels when compared to HP women with the same haplotype. While previous studies in cardiovascular and metabolic diseases have linked specific *ADIPOQ* haplotypes to altered circulating adiponectin levels (Smetnev et al., 2019), their role in HDP remains underexplored. Our findings suggest that, despite inconsistencies across populations, rs266729 SNP may influence plasma adiponectin modulation in PE, potentially reflecting a specific effect distinct from other metabolic or cardiovascular phenotypes.

Finally, the analysis of upper and lower subgroups of plasma adiponectin levels revealed that the ‘G,T,G’ haplotype was more frequent in the subgroup of higher adiponectin levels in PE. However, the ‘G,T,G’ haplotype was not associated with PE or GH in the overall haplotype distribution. As aforementioned, the ‘C,T,T’ haplotype was more frequent in HP than GH. Consistent with this finding, the ‘C,T,T’ haplotype was more frequent in the lower subgroup of adiponectin levels in PE. Taken together, we suggest that this haplotype may be associated with adiponectin levels and the outcome of HDP. Nonetheless, further studies are needed to explore this hypothesis. These findings are also aligned with the proposal that comparing extremes of phenotype distribution improves our abilities to find genetic contributions to phenotypes (Metzger et al., 2013; Nebert, 2000).

This study has limitations. First, the number of patients included in the study was relatively small, with an even smaller subset having plasma samples available to measure adiponectin levels, which limits the statistical power for this subgroup analysis on both adjusted and unadjusted analysis. Nevertheless, we identified significant associations involving genotypes and haplotypes of *ADIPOQ* with PE and GH, especially involving SNP rs266729. Furthermore, we did not assess adiponectin levels in placental or adipose tissues, leaving the extent of the SNPs effects at the tissue level unknown. In conclusion, we found that the GG genotype for the *ADIPOQ* SNP rs266729 (C>G) was associated with risk for GH, whereas the CG genotype and the ‘C,T,T’ haplotype may protect against PE and GH, respectively. In addition, CG and GG genotypes for the same SNP as well as the ‘G,T,G’ haplotype showed higher plasma adiponectin levels in PE subjects.

## 5 Conclusion

In this study, we provide evidence that the *ADIPOQ* genotypes and haplotypes studied may affect circulating adiponectin in PE and affect the susceptibility to GH or PE. Regarding rs266729 (C>G) SNP, the GG genotype was associated with risk for GH, and the CG genotype may protect against PE. PE patients carrying the CG and GG genotypes showed higher adiponectin levels than their CC counterparts. Regarding haplotypes formed by three *ADIPOQ* SNPs, the ‘G,T,G’ haplotype showed higher adiponectin levels in PE than in HP, and the ‘C,T,T’ haplotype may protect against GH. These findings suggest that *ADIPOQ* polymorphisms influence circulating adiponectin levels and an association with susceptibility for development of GH and PE.

## Data Availability

The original contributions presented in the study are included in the article/Supplementary Material, further inquiries can be directed to the corresponding author.
