# Supplementary material for: ADIPOQ polymorphisms and haplotypes affect circulating adiponectin levels and their association with gestational hypertension and preeclampsia
Source: Front Physiol. 2026 Jan 12;16:1736993. doi: 10.3389/fphys.2025.1736993 (PMC12832239; doi:10.3389/fphys.2025.1736993)
Supplement: Supplementary file 1 [file Table1.docx]

**Supplementary Table 1.** Clinical and demographic characteristics for subjects whose plasma samples were available.

| **Parameters** | **HP (n = 130)** | **GH (n = 42)** | **P value^a^** | **PE (n = 55)** | **P value^a^** |
| --- | --- | --- | --- | --- | --- |
| Age (years) | 24.00 (20.00 - 29.00) | 27.50 (21.25 - 33.00) | 0.0563 | 26.00 (20.25 - 32.75) | 0.1593 |
| Race (% white) | 63.00 | 71.42 | 0.3555 | 63.63 | >0.9999 |
| Current smoking (%) | 10.70 | 16.70 | 0.4151 | 7.27 | 0.5924 |
| Primiparity (%) | 44.61 | 40.47 | 0.7215 | 43.63 | >0.9999 |
| BMI (kg/m^2^) | 27.05 (24.68 - 30.00) | 29.83 (27.14 - 33.11) | **0.0002** | 29.13 (26.11 - 32.01) | **0.0029** |
| Pre-pregnancy BMI (kg/m^2^) | 22.20 (17.50 - 26.60) | 30.06 (17.31 - 49.61) | **<0.0001** | 25.15 (16.18 - 48.41) | **0.0010** |
| SBP (mm Hg) | 110.00 (101.50 - 120.00) | 130.00 (123.50 - 140.00) | **<0.0001** | 140.00 (131.00 - 155.00) | **<0.0001** |
| DBP (mm Hg) | 70.00 (70.00 - 80.00) | 84.00 (73.00 - 90.00) | **<0.0001** | 90.00 (80.00 - 100.00) | **<0.0001** |
| HR (beats/min) | 80.00 (80.00 - 86.00) | 82.00 (80.00 - 87.00) | 0.9197 | 80.00 (80.00 - 87.50) | 0.7698 |
| Fasting glucose (mg/dL) | 73.00 (66.00 - 82.00) | 76.00 (69.00 - 87.50) | 0.3965 | 80.00 (72.63 - 97.50) | **0.0029** |
| Hb (g/dL) | 12.00 (11.00 - 12.75) | 11.90 (11.20 - 12.70) | 0.5335 | 12.40 (11.70 - 13.05) | 0.0646 |
| Hct (%) | 35.85 (33.00 - 38.15) | 35.95 (33.48 - 37.90) | 0.8163 | 37.00 (34.50 - 39.50) | **0.0287** |
| Creatinine (mmol/L) | 0.60 (0.60 - 0.80) | 0.60 (0.50 - 0.70) | 0.1574 | 0.70 (0.60 - 0.80) | 0.8952 |
| 24 h Pr (mg/24 h) | ND | 146.00 (110.00 - 166.50) | ND | 726.20 (302.80 - 1777) | ND |
| GAS (weeks) | 37.00 (36.00 - 38.00) | 37.00 (36.00 - 38.00) | 0.9397 | 35.00 (32.00 - 37.00) | **<0.0001** |
| GAD (weeks) | 40.00 (39.00 - 41.00) | 39.00 (38.00 - 39.50) | **0.0133** | 36.50 (34.00-38.25) | **<0.0001** |
| Newborn weight (g) | 3263 (2974 - 3681) | 3040 (2833 - 3295) | **0.0258** | 2563 (1665 - 3156) | **<0.0001** |

Abbreviations: BMI, body mass index; DBP, diastolic blood pressure; GAD, gestational age at delivery; GAS, gestational age at sampling; GH, gestational hypertension; Hb, hemoglobin concentration; Hct, hematocrit; HP, healthy pregnant; HR, heart rate; ND, not determined; PE, preeclampsia; SBP, systolic blood pressure. Values are the mean ± s.e.m. ^a^ P < 0.05 vs healthy pregnant group. Bold values are significant.

**Supplementary Table 2**. Generalized linear model (GLM - Gamma) for HP compared to PE, considering adiponectin levels as the dependent variable.

| ***GLM Adiponectin levels*** | **Estimate** | **Std. Error** | **Statistic** | **P value** | **Ratio of Means (95% CI)** |
| --- | --- | --- | --- | --- | --- |
| *(Intercept)* | 15,5150 | 0,8772 | 3,1256 | **0,0028** | 15.5150 (2.6055 – 96.1052) |
| Group (PE) | 1,2019 | 0,1303 | 1,4115 | 0,1636 | 1.2019 (0.9217 – 1.5761) |
| BMI (Kg/m²) | 1,0029 | 0,0133 | 0,2180 | 0,8282 | 1.0029 (0.9780 – 1.0295) |
| GAS (weeks) | 1,0094 | 0,0200 | 0,4675 | 0,6419 | 1.0094 (0.9667 – 1.0526) |
| Fasting glucose (mg/dL) | 0,9966 | 0,0053 | -0,6412 | 0,5240 | 0.9966 (0.9863 – 1.0072) |
| Ethnicity  mixed | 1,1093 | 0,1365 | 0,7601 | 0,4504 | 1.1093 (0.8571 – 1.4536) |
| Ethnicity  black | 0,7664 | 0,1386 | -1,9194 | 0,0600 | 0.7664 (0.5881 – 1.0122) |

BMI, Body mass index; CI, confidence intervals; GAS, gestational age at sampling; PE, preeclampsia. Significant *P* values are bold. Observations included in model: N = 63 (GS = 41; PE = 22); AIC = 93.39

**Supplementary Table 3.** Haplotype frequencies for ADIPOQ polymorphisms in the healthy pregnant group segregated according to lower and upper plasma levels of adiponectin.

| ***Haplotypes*** | ***Hap-Score*** | ***Lower (n = 2 x 66)*** | ***Upper (n = 2 x 64)*** | ***P value*** | ***OR (95% CI)*** |
| --- | --- | --- | --- | --- | --- |
| C,T,G | 0.9312 | 0.4669 (46,69%) | 0.3497 (34,97%) | 0.3517 | 1.0000 (*Reference*) |
| C,T,T | -0.4257 | 0.2359 (23,59%) | 0.2926 (29,26%) | 0.6703 | 0.6653 (0.3353 - 1.3200) |
| G,T,G | -0.8186 | 0.1164 (11,64%) | 0.2128 (21,28%) | 0.4130 | 0.4264 (0.1580 - 1.1513) |
| C,G,G | -1.0109 | 0.0472 (4,72%) | 0.1077 (10,77%) | 0.3121 | 0.4403 (0.1320 - 1.4693) |
| Global-stat = 5.4738. df = 5; P = 0.3608 | | | | | |

Abbreviations: CI, Confidence Interval; OR, Odds Ratio.

**Supplementary Table 4** Haplotype frequencies for ADIPOQ polymorphisms in the gestational hypertension group segregated according to lower and upper plasma levels of adiponectin.

| ***Haplotypes*** | ***Hap-Score*** | ***Lower (n = 2 x 21)*** | ***Upper (n = 2x 21)*** | ***P value*** | ***OR (95% CI)*** |
| --- | --- | --- | --- | --- | --- |
| C,T,G | -1.7110 | 0.2403 (24,03%) | 0.4454 (44,54%) | 0.0871 | 1.0000 (*Reference*) |
| C,T,T | 0.9394 | 0.2597 (25,97%) | 0.1499 (14,99%) | 0.3475 | 2.8999 (0.7914 - 10.6260) |
| G,T,G | 0.0460 | 0.2359 (23,59%) | 0.2213 (22,13%) | 0.9633 | 1.6734 (0.4762 - 5.8810) |
| C,G,G | 0.7716 | 0.1905 (19,05%) | 0.1190 (11,90%) | 0.4403 | 3.8587 (0.8139 - 18.2900) |
| Global-stat = 6.3497. df = 6. P = 0.3852 | | | | | |

Abbreviations: CI, Confidence Interval; OR, Odds Ratio.

**Supplementary Table 5.** Haplotypes frequencies for ADIPOQ polymorphisms in the preeclampsia group segregated according to lower and upper levels of plasma adiponectin.

| ***Haplotypes*** | ***Hap-Score*** | ***Lower (n = 2 x 28)*** | ***Upper (n = 2 x 27)*** | ***P value*** | ***OR (95% CI)*** |
| --- | --- | --- | --- | --- | --- |
| C,T,G | 0.4035 | 0.3571 (35,71%) | 0.3380 (33,80%) | 0.6865 | 1.0000 (*Reference*) |
| C,T,T | 2.1905 | 0.3929 (39,29%) | 0.1805 (18,05%) | **0.0285** | 1.8035 (0.6678 - 4.871) |
| C,G,G | 0.4148 | 0.1428 (14,28%) | 0.1111 (11,11%) | 0.6783 | 1.0252 (0.2678 - 3.925) |
| G,T,G | -2.0770 | 0.1071 (10,71%) | 0.2546 (25,46%) | **0.0378** | 0.5651 (0.1491 - 2.1420) |

Global-stat = 8.8209. df = 5. P = 0.1164

Abbreviations: CI, Confidence Interval; OR, Odds Ratio. Significant P values are in bold.

**Supplementary Table 6.** Gestational Hypertension (GH) univariate logistic regression.

| ***Logistic Model GH*** | ***Estimate*** | ***Std. Error z*** | ***z value*** | ***Pr(>\|z\|)*** | ***OR (95% CI)*** |
| --- | --- | --- | --- | --- | --- |
| *(Intercept)* | -6.2472 | 0.8994 | -6.9459 | **< 0.0001** | 0.0019 (0.0003 - 0.0104) |
| *BMI (Kg/m²) during pregnancy* | 0.1939 | 0.0295 | 6.5662 | **< 0.0001** | 1.2140 (1.1491 - 1.2905) |
| *(Intercept)* | -1.7537 | 0.5207 | -3.3682 | **0.0008** | 0.1731 (0.0611 - 0.4732) |
| *Age (Years)* | 0.053 | 0.0194 | 2.737 | **0.0062** | 1.0544 (1.0156 - 1.096) |
| *(Intercept)* | 1.1034 | 1.2454 | 0.886 | 0.3756 | 3.0144 (0.2681 - 37.7634) |
| *GAS (weeks)* | -0.0325 | 0.034 | -0.9555 | 0.3393 | 0.9680 (0.9036 - 1.0341) |
| *(Intercept)* | -0.1201 | 0.1417 | -0.848 | 0.3964 | 0.8868 (0.6710 - 1.1702) |
| *Ethnicity (brown)* | -0.642 | 0.3533 | -1.817 | 0.0692 | 0.5262 (0.2567 - 1.035) |
| *Ethnicity (black)* | -0.2593 | 0.3868 | -0.6705 | 0.5026 | 0.7716 (0.3544 - 1.6345) |
| *(Intercept)* | -0.2032 | 0.1599 | -1.2707 | 0.2038 | 0.8161 (0.5951 - 1.1154) |
| *Primiparity* | -0.2022 | 0.2427 | -0.8332 | 0.4047 | 0.8169 (0.5065 - 1.3131) |
| *(Intercept)* | -0.5046 | 0.1583 | -3.1873 | **0.0014** | 0.6038 (0.4407 - 0.8207) |
| *rs266729CG* | -0.0117 | 0.2612 | -0.0446 | 0.9644 | 0.9884 (0.5901 - 1.6458) |
| *rs266729GG* | 0.8612 | 0.3827 | 2.2502 | **0.0244** | 2.3661 (1.1254 - 5.0979) |
| *(Intercept)* | 1.0986 | 1.1547 | 0.9514 | 0.3414 | 3.0000 (0.3841 - 60.6487) |
| *rs2241766TG* | -1.2528 | 1.1812 | -1.0606 | 0.2889 | 0.2857 (0.0137 - 2.3639) |
| *rs2241766TT* | -1.6049 | 1.1626 | -1.3805 | 0.1674 | 0.2009 (0.0099 - 1.5963) |
| *(Intercept)* | -0.2201 | 0.1665 | -1.3215 | 0.1863 | 0.8025 (0.5775 - 1.1108) |
| *rs1501299GT* | -0.3365 | 0.2474 | -1.3602 | 0.1738 | 0.7143 (0.4385 - 1.1581) |
| *rs1501299TT* | -0.5272 | 0.4376 | -1.2047 | 0.2283 | 0.5903 (0.2402 - 1.3595) |

**Supplementary Table 7.** Preeclampsia (PE) univariate logistic regression.

| ***Logistic Model PE*** | ***Estimate*** | ***Std. Error z*** | ***z value*** | ***Pr(>\|z\|)*** | ***OR (95% CI)*** |
| --- | --- | --- | --- | --- | --- |
| *(Intercept)* | -4.9106 | 0.8052 | -6.0983 | **< 0.0001** | 0.0074 (0.0014 - 0.0335) |
| *BMI (Kg/m²) during pregnancy* | 0.1587 | 0.0268 | 5.9095 | **< 0.0001** | 1.1719 (1.1145 - 1.2386) |
| *(Intercept)* | -1.6228 | 0.5144 | -3.155 | **0.0016** | 0.1973 (0.0706 - 0.5331) |
| *Age (Years)* | 0.0554 | 0.0192 | 2.8798 | **0.0040** | 1.057 (1.0184 - 1.0984) |
| *(Intercept)* | 5.0668 | 1.3634 | 3.7163 | **0.0002** | 158.6716 (12.9145 - 2764.8681) |
| *GAS (weeks)* | -0.1406 | 0.0376 | -3.7348 | **0.0002** | 0.8688 (0.8031 - 0.9314) |
| *(Intercept)* | 0 | 0.1443 | 0 | 1.0000 | 1.0000 (0.7532 - 1.3276) |
| *Ethnicity (brown)* | -0.3514 | 0.3324 | -1.0571 | 0.2905 | 0.7037 (0.3626 - 1.3433) |
| *Ethnicity (black)* | -0.1112 | 0.3637 | -0.3058 | 0.7598 | 0.8947 (0.4351 - 1.8273) |
| *(Intercept)* | -0.0892 | 0.1598 | -0.5585 | 0.5765 | 0.9146 (0.6678 - 1.2509) |
| *Primiparity* | -0.1049 | 0.241 | -0.4353 | 0.6633 | 0.9004 (0.5606 - 1.4437) |
| *(Intercept)* | -0.1231 | 0.1498 | -0.8217 | 0.4113 | 0.8842 (0.6583 - 1.1855) |
| *rs266729CG* | -0.6223 | 0.274 | -2.2708 | **0.0232** | 0.5367 (0.3107 - 0.9121) |
| *rs266729GG* | 0.5538 | 0.3865 | 1.4331 | 0.1518 | 1.7399 (0.8233 - 3.7893) |
| *(Intercept)* | 1.6094 | 1.0954 | 1.4692 | 0.1418 | 5.0000 (0.8064 - 95.7954) |
| *rs2241766TG* | -1.7476 | 1.1266 | -1.5512 | 0.1209 | 0.1742 (0.0088 - 1.1681) |
| *rs2241766TT* | -1.9095 | 1.1034 | -1.7307 | 0.0835 | 0.1481 (0.0077 - 0.9372) |
| *(Intercept)* | -0.0858 | 0.1692 | -0.5069 | 0.6122 | 0.9178 (0.6578 - 1.2787) |
| *rs1501299GT* | -0.2999 | 0.2481 | -1.2087 | 0.2268 | 0.7409 (0.4545 - 1.2035) |
| *rs1501299TT* | -0.2196 | 0.3907 | -0.562 | 0.5741 | 0.8028 (0.3677 - 1.7189) |

**Supplementary Table 8.** Gestational Hypertension (GH) multivariate logistic regression.

| ***Logistic Model GH*** | ***Estimate*** | ***Std. Error z*** | ***z value*** | ***Pr(>\|z\|)*** | ***OR (95% CI)*** |
| --- | --- | --- | --- | --- | --- |
| *(Intercept)* | -6.7643 | 1.0407 | -6.4996 | **< 0.0001** | 0.0012 (0.0001 - 0.0081) |
| *rs266729CG* | -0.1070 | 0.3112 | -0.3437 | 0.7311 | 0.8986 (0.4848 - 1.6478) |
| *rs266729GG* | 0.4082 | 0.4625 | 0.8824 | 0.3775 | 1.5040 (0.6070 - 3.7693) |
| *Age (years)* | 0.0368 | 0.0222 | 1.6581 | 0.0973 | 1.0374 (0.9934 - 1.0840) |
| *BMI (Kg/m²) during pregnancy* | 0.1776 | 0.0295 | 6.0136 | **< 0.0001** | 1.1944 (1.1304 - 1.2697) |
| *(Intercept)* | -6.1319 | 0.9006 | -6.8085 | **< 0.0001** | 0.0022 (0.0003 - 0.0116) |
| *rs266729CG* | -0.1237 | 0.3085 | -0.401 | 0.6884 | 0.8836 (0.4792 - 1.612) |
| *rs266729GG* | 0.3884 | 0.4555 | 0.8526 | 0.3939 | 1.4746 (0.6039 - 3.6473) |
| *BMI (Kg/m²) during pregnancy* | 0.1899 | 0.0296 | 6.4094 | **< 0.0001** | 1.2092 (1.1443 - 1.2857) |
| *(Intercept)* | -1.8839 | 0.5330 | -3.5343 | **0.0004** | 0.1520 (0.0522 - 0.4247) |
| *rs266729CG* | 0.0216 | 0.2737 | 0.0789 | 0.9371 | 1.0218 (0.5951 - 1.7436) |
| *rs266729GG* | 0.9140 | 0.4144 | 2.2056 | **0.0274** | 2.4943 (1.1173 - 5.7440) |
| *Age (years)* | 0.0539 | 0.0197 | 2.7380 | **0.0062** | 1.0553 (1.0159 - 1.0977) |
| *(Intercept)* | -5.5939 | 1.5834 | -3.5329 | **0.0004** | 0.0037 (0.0002 - 0.1247) |
| *rs2241766TG* | -0.9645 | 1.2544 | -0.7688 | 0.4420 | 0.3812 (0.0170 - 3.8723) |
| *rs2241766TT* | -1.3663 | 1.2228 | -1.1173 | 0.2639 | 0.2551 (0.0118 - 2.4217) |
| *Age (years)* | 0.0352 | 0.0224 | 1.572 | 0.116 | 1.0358 (0.9914 - 1.0828) |
| *BMI (Kg/m²) during pregnancy* | 0.1822 | 0.0298 | 6.1181 | **< 0.0001** | 1.1999 (1.1350 - 1.2761) |
| *(Intercept)* | -5.2258 | 1.5266 | -3.4232 | **0.0006** | 0.0054 (0.0003 - 0.1670) |
| *rs2241766TG* | -0.7064 | 1.2478 | -0.5661 | 0.5713 | 0.4934 (0.0222 - 5.0167) |
| *rs2241766TT* | -1.1699 | 1.2215 | -0.9578 | 0.3382 | 0.3104 (0.0144 - 2.9859) |
| *BMI (Kg/m²) during pregnancy* | 0.1950 | 0.0298 | 6.5463 | **< 0.0001** | 1.2153 (1.1497 - 1.2924) |
| *(Intercept)* | -0.0132 | 1.2279 | -0.0107 | 0.9914 | 0.9869 (0.1068 - 21.6036) |
| *rs2241766TG* | -1.6379 | 1.2009 | -1.3639 | 0.1726 | 0.1944 (0.0091 - 1.6741) |
| *rs2241766TT* | -1.8497 | 1.1753 | -1.5738 | 0.1155 | 0.1573 (0.0076 - 1.2828) |
| *Age (years)* | 0.0544 | 0.0198 | 2.7480 | **0.0060** | 1.0559 (1.0162 - 1.0985) |
| *(Intercept)* | -6.7060 | 1.0469 | -6.4059 | **< 0.0001** | 0.0012 (0.0001 - 0.0087) |
| *rs1501299GT* | -0.4331 | 0.2983 | -1.4521 | 0.1465 | 0.6485 (0.3589 - 1.1589) |
| *rs1501299TT* | -0.4977 | 0.5449 | -0.9133 | 0.3611 | 0.6080 (0.1975 - 1.7112) |
| *Age (years)* | 0.0371 | 0.0220 | 1.6843 | 0.0921 | 1.0378 (0.9940 - 1.0840) |
| *BMI (Kg/m²) during pregnancy* | 0.1831 | 0.0297 | 6.1670 | **< 0.0001** | 1.2009 (1.1363 - 1.2770) |
| *(Intercept)* | -6.0642 | 0.9052 | -6.6990 | **< 0.0001** | 0.0023 (0.0004 - 0.0126) |
| *rs1501299GT* | -0.4709 | 0.2958 | -1.5921 | 0.1114 | 0.6244 (0.3472 - 1.1104) |
| *rs1501299TT* | -0.3893 | 0.5227 | -0.7447 | 0.4565 | 0.6776 (0.2321 - 1.8386) |
| *BMI (Kg/m²) during pregnancy* | 0.1953 | 0.0297 | 6.5772 | **< 0.0001** | 1.2157 (1.1504 - 1.2928) |
| *(Intercept)* | -1.6181 | 0.5348 | -3.0257 | **0.0025** | 0.1983 (0.0682 - 0.5578) |
| *rs1501299GT* | -0.2407 | 0.2588 | -0.9301 | 0.3523 | 0.7861 (0.4721 - 1.3040) |
| *rs1501299TT* | -0.4729 | 0.4707 | -1.0045 | 0.3151 | 0.6232 (0.2366 - 1.5310) |
| *Age (years)* | 0.0532 | 0.0194 | 2.7387 | **0.0062** | 1.0547 (1.0158 - 1.0965) |

Abbreviations: BMI, Body mass index; CI, confidence intervals; OR, odds ratio; GH, gestational hypertension. Significant P values are in bold.

**Supplementary Table 9.** Preeclampsia (PE) multivariate logistic regression.

| ***Logistic Model PE*** | ***Estimate*** | ***Std. Error z*** | ***z value*** | ***Pr(>\|z\|)*** | ***OR (95% CI)*** |
| --- | --- | --- | --- | --- | --- |
| *(Intercept)* | -5.6026 | 0.9455 | -5.9255 | **< 0.0001** | 0.0037 (0.0005 - 0.0220) |
| *rs266729CG* | -0.6548 | 0.3105 | -2.1090 | **0.0349** | 0.5195 (0.2794 - 0.9471) |
| *rs266729GG* | 0.2618 | 0.4591 | 0.5702 | 0.5686 | 1.2992 (0.5296 - 3.2518) |
| *Age (years)* | 0.0449 | 0.0213 | 2.1073 | **0.0351** | 1.0459 (1.0036 - 1.0912) |
| *BMI (Kg/m²) during pregnancy* | 0.1465 | 0.0275 | 5.3197 | **< 0.0001** | 1.1578 (1.0995 - 1.2253) |
| *(Intercept)* | -4.6682 | 0.8164 | -5.7184 | **< 0.0001** | 0.0094 (0.0018 - 0.0437) |
| *rs266729CG* | -0.6063 | 0.3027 | -2.0032 | **0.0452** | 0.5454 (0.2982 - 0.9801) |
| *rs266729GG* | 0.2705 | 0.4459 | 0.6066 | 0.5441 | 1.3106 (0.5494 - 3.2028) |
| *BMI (Kg/m²) during pregnancy* | 0.1554 | 0.0272 | 5.7063 | **< 0.0001** | 1.1681 (1.1100 - 1.2355) |
| *(Intercept)* | -1.5974 | 0.5284 | -3.0233 | **0.0025** | 0.2024 (0.0704 - 0.5620) |
| *rs266729CG* | -0.6797 | 0.2881 | -2.3589 | **0.0183** | 0.5068 (0.2850 - 0.8845) |
| *rs266729GG* | 0.6093 | 0.4158 | 1.4654 | 0.1428 | 1.8392 (0.8252 - 4.2718) |
| *Age (years)* | 0.0592 | 0.0197 | 3.0002 | **0.0027** | 1.0610 (1.0213 - 1.1038) |
| *(Intercept)* | -3.8556 | 1.4515 | -2.6563 | **0.0079** | 0.0212 (0.0013 - 0.5975) |
| *rs2241766TG* | -1.9015 | 1.2587 | -1.5107 | 0.1309 | 0.1493 (0.0066 - 1.4532) |
| *rs2241766TT* | -2.1229 | 1.2339 | -1.7205 | 0.0853 | 0.1197 (0.0054 - 1.1006) |
| *Age (years)* | 0.0435 | 0.0210 | 2.0699 | **0.0385** | 1.0444 (1.0027 - 1.0890) |
| *BMI (Kg/m²) during pregnancy* | 0.1524 | 0.0275 | 5.5401 | **< 0.0001** | 1.1646 (1.1062 - 1.2326) |
| *(Intercept)* | -3.1721 | 1.4093 | -2.2508 | **0.0244** | 0.0419 (0.0029 - 1.1213) |
| *rs2241766TG* | -1.7039 | 1.2578 | -1.3547 | 0.1755 | 0.1820 (0.0080 - 1.7502) |
| *rs2241766TT* | -1.8470 | 1.2315 | -1.4998 | 0.1337 | 0.1577 (0.0072 - 1.4282) |
| *BMI (Kg/m²) during pregnancy* | 0.1605 | 0.0272 | 5.9042 | **< 0.0001** | 1.1741 (1.1159 - 1.2418) |
| *(Intercept)* | 0.3505 | 1.1710 | 0.2993 | 0.7647 | 1.4198 (0.1876 - 29.4092) |
| *rs2241766TG* | -1.9896 | 1.1424 | -1.7416 | 0.0816 | 0.1368 (0.0068 - 0.9507) |
| *rs2241766TT* | -2.2134 | 1.1197 | -1.9768 | **0.0481** | 0.1093 (0.0056 - 0.7181) |
| *Age (years)* | 0.0613 | 0.0196 | 3.1277 | **0.0018** | 1.0632 (1.0237 - 1.1057) |
| *(Intercept)* | -5.6971 | 0.9471 | -6.0151 | **< 0.0001** | 0.0034 (0.0005 - 0.0200) |
| *rs1501299GT* | -0.0674 | 0.2825 | -0.2384 | 0.8116 | 0.9349 (0.5368 - 1.6286) |
| *rs1501299TT* | -0.1232 | 0.4569 | -0.2695 | 0.7875 | 0.8841 (0.3553 - 2.1563) |
| *Age (years)* | 0.0394 | 0.0208 | 1.8912 | **0.0586** | 1.0401 (0.9989 - 1.0841) |
| *BMI (Kg/m²) during pregnancy* | 0.1505 | 0.0272 | 5.5366 | **< 0.0001** | 1.1624 (1.1047 - 1.2293) |
| *(Intercept)* | -4.8163 | 0.8210 | -5.8662 | **< 0.0001** | 0.0081 (0.0015 - 0.0380) |
| *rs1501299GT* | -0.1557 | 0.2776 | -0.5609 | 0.5749 | 0.8558 (0.4957 - 1.4753) |
| *rs1501299TT* | -0.0889 | 0.4439 | -0.2002 | 0.8413 | 0.9150 (0.3780 - 2.1794) |
| *BMI (Kg/m²) during pregnancy* | 0.1580 | 0.0269 | 5.8786 | **< 0.0001** | 1.1712 (1.1137 - 1.2378) |
| *(Intercept)* | -1.5266 | 0.5317 | -2.8710 | **0.0041** | 0.2173 (0.0752 - 0.6079) |
| *rs1501299GT* | -0.1763 | 0.2584 | -0.6824 | 0.4950 | 0.8384 (0.5044 - 1.3907) |
| *rs1501299TT* | -0.1547 | 0.4174 | -0.3708 | 0.7108 | 0.8566 (0.3727 - 1.9378) |
| *Age (years)* | 0.0552 | 0.0193 | 2.8622 | **0.0042** | 1.0568 (1.0181 - 1.0983) |

Abbreviations: BMI, Body mass index; CI, confidence intervals; OR, odds ratio; PE, preeclampsia. Significant *P* values are in bold.

# **Supplementary Material 1.** Results for Hardy-Weinberg Equilibrium

File: SNPs of *ADIPOQ* genes

Number of populations detected: 3

Number of loci detected: 3

Estimation of exact P-Values by the Markov chain method.

---------------------------------------------

Markov chain parameters for all tests:

Dememorization: 1000

Batches: 100

Iterations per batch: 1000

Hardy Weinberg: Probability test

************************

==========================================

Results by population

==========================================

Pop : HP

-----------------------------------------

Fis estimates

---------------

locus P-val S.E. W&C R&H Steps

----------- ------- ------- ------- ------- ------

rs266729 0.6463 0.0036 0.0398 0.0399 81382 switches

rs2241766 1.0000 0.0000 -0.0140 -0.0141 60845 switches

rs1501299 0.4351 0.0048 -0.0734 -0.0737 84546 switches

All (Fisher's method):

Chi2 : 2.5375

Df : 6

Prob : 0.864248

Pop : GH

-----------------------------------------

Fis estimates

---------------

locus P-val S.E. W&C R&H Steps

----------- ------- ------- ------- ------- ------

rs266729 0.1394 0.0029 0.1895 0.1911 76599 switches

rs2241766 1.0000 0.0000 0.0258 0.0260 67692 switches

rs1501299 0.5325 0.0025 0.0959 0.0967 76418 switches

All (Fisher's method):

Chi2 : 5.2010

Df : 6

Prob : 0.518303

Pop : PE

-----------------------------------------

Fis estimates

---------------

locus P-val S.E. W&C R&H Steps

----------- ------- ------- ------- ------- ------

rs266729 0.0009 0.0002 0.3632 0.3660 77462 switches

rs2241766 0.0678 0.0018 0.2114 0.2129 67989 switches

rs1501299 0.8078 0.0018 0.0404 0.0407 80931 switches

All (Fisher's method):

Chi2 : 19.7921

Df : 6

Prob : 0.003015

==========================================

All locus, all populations

==========================================

All (Fisher's method) :

Chi2 : 27.5306

Df : 18

Prob : 0.069566
