# Supplementary material for: ADIPOQ polymorphisms and haplotypes affect circulating adiponectin levels and their association with gestational hypertension and preeclampsia
Source: Front Physiol. 2026 Jan 12;16:1736993. doi: 10.3389/fphys.2025.1736993 (PMC12832239; doi:10.3389/fphys.2025.1736993)
Supplement: Supplementary file 2 [file DataSheet1.docx]

**Supplementary Figure 1**

**
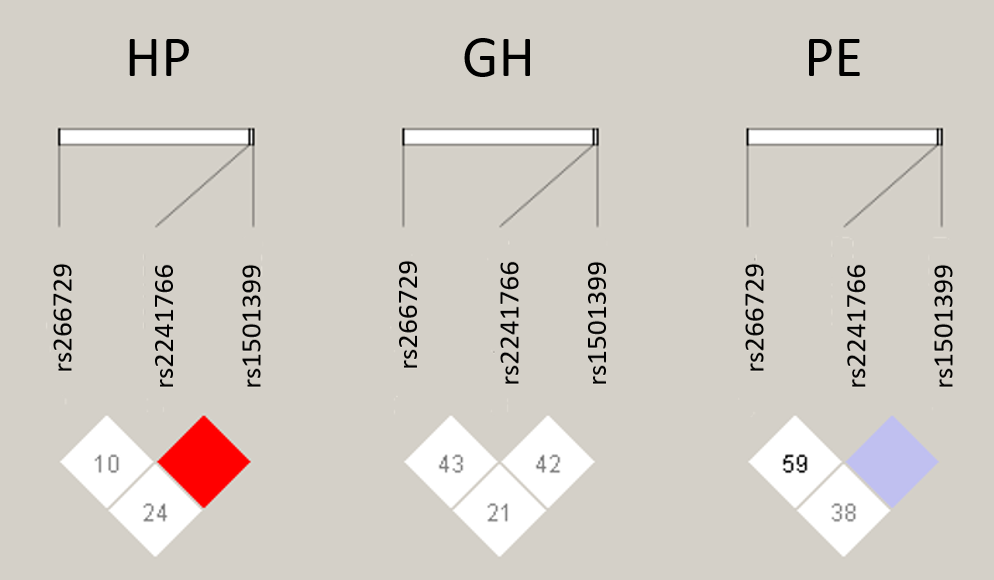
**

**Supplementary Figure 1.** Linkage disequilibrium plots for the *ADIPOQ* SNPs in healthy pregnant (HP), gestational hypertension (GH), and preeclampsia (PE) groups. Values for pairwise D ́ are presented in each box; those without values refer to D=1. Color scheme: bright red, D=1 and LOD≥2; blue, D=1 and LOD<2; white, D<1 and LOD<2. LOD, Logarithm of odds.

**Supplementary Figure 2**


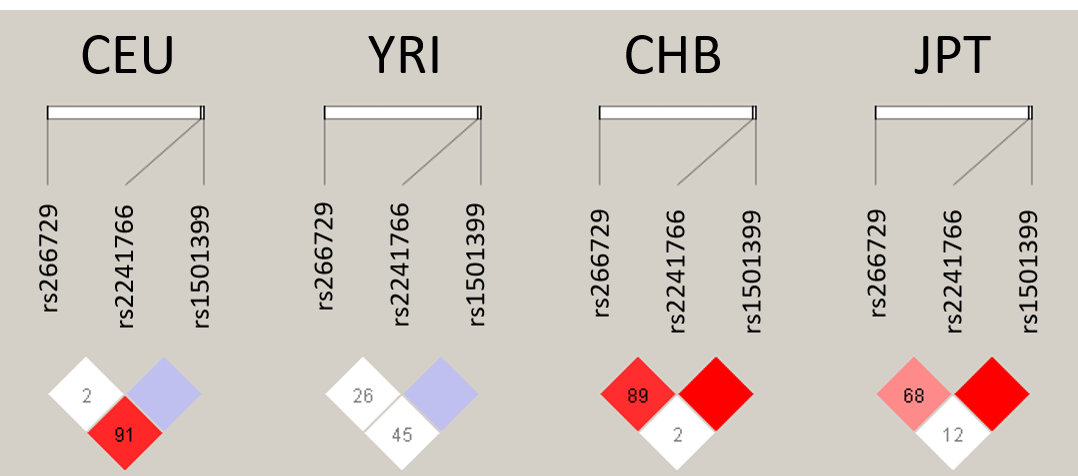


**Supplementary Figure 2.** Linkage disequilibrium plots for the ADIPOQ SNPs in four different populations of the 1000 Genomes Project: European (CEU), African (YRI), Chinese (CHB), and Japanese (JPT). Values for pairwise D ́ are presented in each box; those without values refer to D=1. Color scheme: bright red, D=1 and LOD≥2; blue, D=1 and LOD<2; white, D<1 and LOD<2. LOD, Logarithm of odds.

**Supplementary Figure 3**


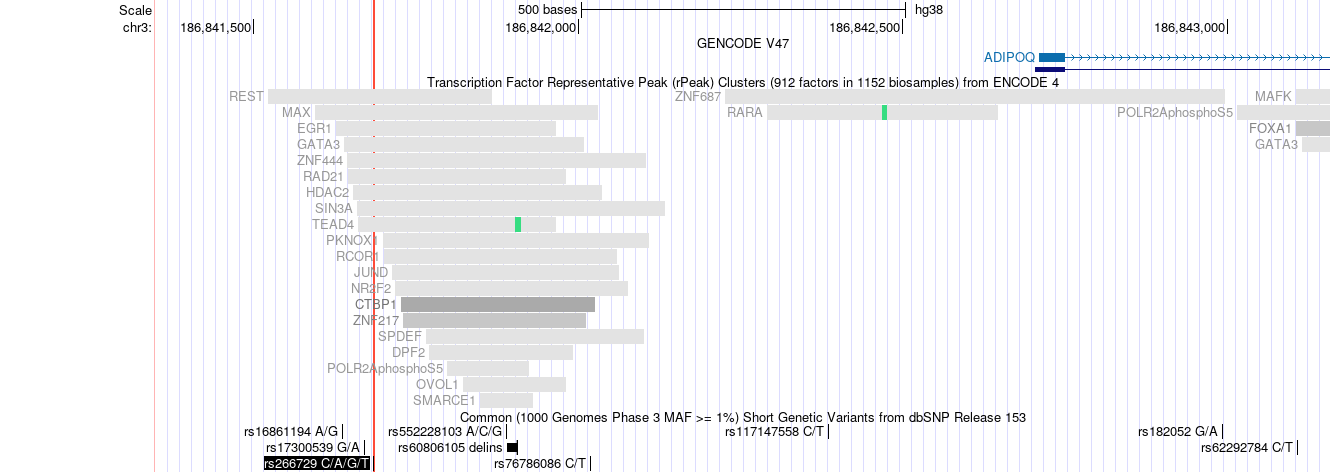


**Supplementary Figure 3.** UCSC Genome Browser view of the ADIPOQ promoter region, with the location of the rs266729 SNP (red highlight) and transcription factor binding sites from the ENCODE data. The region shows an abundance transcription factor binding sites located in the SNP site and nearby.
